# A Quantitative Analysis of Flight Feather Replacement in the Moustached Tree Swift *Hemiprocne mystacea*, a Tropical Aerial Forager

**DOI:** 10.1371/journal.pone.0011586

**Published:** 2010-07-14

**Authors:** Sievert Rohwer, Luan-Keng Wang

**Affiliations:** Burke Museum and Department of Biology, University of Washington, Seattle, Washington, United States of America; University of Bristol, United Kingdom

## Abstract

The functional life span of feathers is always much less than the potential life span of birds, so feathers must be renewed regularly. But feather renewal entails important energetic, time and performance costs that must be integrated into the annual cycle. Across species the time required to replace flight feather increases disproportionately with body size, resulting in complex, multiple waves of feather replacement in the primaries of many large birds. We describe the rules of flight feather replacement for *Hemiprocne mystacea*, a small, 60g tree swift from the New Guinea region. This species breeds and molts in all months of the year, and flight feather molt occurs during breeding in some individuals. *H. mystacea* is one to be the smallest species for which stepwise replacement of the primaries and secondaries has been documented; yet, primary replacement is extremely slow in this aerial forager, requiring more than 300 days if molt is not interrupted. We used growth bands to show that primaries grow at an average rate of 2.86 mm/d. The 10 primaries are a single molt series, while the 11 secondaries and five rectrices are each broken into two molt series. In large birds stepwise replacement of the primaries serves to increase the rate of primary replacement while minimizing gaps in the wing. But stepwise replacement of the wing quills in *H. mystacea* proceeds so slowly that it may be a consequence of the ontogeny of stepwise molting, rather than an adaptation, because the average number of growing primaries is probably lower than 1.14 feathers per wing.

## Introduction

The feathers of birds have a functional life span that is always much shorter than the potential life span of adult birds. Thus feathers must be renewed regularly during periods of molt, wherein old feathers are pushed out and lost as new feathers are generated from the same feather follicle. Molt is costly to birds because feathers are time-consuming to build, because the synthesis of new feathers results in the turnover of a great deal of body protein, and because molt gaps in the flight feathers reduce lift and missing or growing contour feathers create irregularities in the body surface that increase drag [1]–[4]. The aerodynamic costs of molt likely also increase the risk of predation [5]–[7]. To mitigate these costs, birds trade feather renewal off with other life history demands, such as breeding and migrating. Molt and breeding or migrating are often segregated in time to reduce their impacts on daily energetic demands and flight performance, especially in small temperate land birds [8] and, when molt temporally overlaps breeding or migrating, fitness conflicts are suggested by prolonged molts of low intensity [9]. In general for small birds the energetic costs of molt are high [1],[2], but in large birds the allometry of feather size and feather growth rate results in molting making large time demands on the annual cycle [10].

Across species, the time required to replace the primaries increases disproportionately with body size, making molt far more time-demanding for large than small birds. This is true because primary length increases with mass (M) as M^0.31^, while the growth rate of the primaries increases as M^0.17^, meaning that large birds take disproportionately (M^0.31^/M^0.17^ = M^0.14^) more time to replace their primaries [10]. To meet the time demands of molting flight feathers, many large birds that fly while molting have multiple waves of feather replacement proceeding through their primaries simultaneously. Multiple waves of primary replacement occur in two ways. In some groups the primaries are divided into two or more replacement series, as in some albatrosses [11],[12], some parrots [13], and falcons [14],[15]. Alternatively, if the primaries are a single molt series, stepwise replacement results in two or more waves of molt proceeding simultaneously, and in the same direction, through the primaries [16]–[18].

Stepwise primary molt, defined as two or more waves of feather replacement moving through the primaries at the same time and in the same direction [16], is the most common way that avian lineages have achieved multiple waves of feather replacement in their primaries. It is well known for large species such as cormorants [19]–[21], large herons [17], large accipitrids [15],[22], and other groups [18]. The wings of various smaller tropical breeders, such as *Ptilinopus* and *Columbina* doves, francolins, and frogmouths, often contain two runs of new primaries that are separated by older primaries (Rohwer and Wang, unpublished). So far as we know, however, no small species featuring a mix of new and old primaries has been studied in enough detail to determine whether primary replacement is stepwise or whether their primaries are divided into two separate molt series. Most of the literature on flight feather replacement lacks quantitative data summaries that allow critical assessments of the mode of flight feather replacement. Yet critical assessment of conclusions requires knowing the number of growing feathers examined for each flight feather locus and knowing the direction of replacement between each adjacent pair of flight feathers [23].

Here we follow the methods proposed by Rohwer [23] to summarize the rules of flight feather replacement for *Hemiprocne mystacea* (Lesson, 1827), the Moustached Tree Swift. This tropical aerial forager breeds just south of the equator in the Moluccas, New Guinea, most of the Bismarck Archipelago, and the Solomon Islands. At about 60g, this is the smallest resident species for which stepwise replacement of the flight feathers has been described. Primary replacement requires over 300 days. Molt and breeding take place in all months of the year and the intensity of molt in the primaries and secondaries is so low that there is seldom more than a single feather growing per wave of molt. All of these features seem designed to allow extensive overlap between molt and breeding and we have records of adults that were molting primaries when they were shot at their nests. Flight feather replacement is likely to be interrupted when the demands of parental care are large. These interruptions generate the mechanism by which multiple waves of feather replacement that characterize stepwise molting are generated because, in all species where the ontogeny of multiple waves has been established, molt reinitiates both where it was arrested and again at primary one [16],[17].

## Methods

### Abbreviations

Throughout this paper primaries, secondaries and rectrices are abbreviated by P, S, and R, respectively. The primaries are numbered from P1, the innermost primary, to P10, the outermost primary. Secondaries are numbered from S1, the outermost secondary, to S11, the innermost secondary. Rectrices are numbered from R1, the innermost rectrix to R5, the outermost rectrix. Feather pairs are symbolized as P1/P2, or P1/S1, *etc*.

### Scoring molt

Repeated examination of living birds in active molt would be the ideal way to determine the rules of flight feather replacement, but such studies generally follow just a few captive birds. Most quantitative studies of molt come from the examination of museum specimens or from individuals captured during the molt and scored for feather replacement. Rohwer [23] provides a detailed overview of the methods we follow to support our conclusions about the rules of flight feather replacement in *H. mystacea*.

LKW scored immature and adult specimens of *H. mystacea* collected throughout their range to provide data on feather age and feather replacement in the primaries, secondaries and rectrices (see Acknowledgments for collections and acronyms). Recently-fledged birds can easily be distinguished from adults by their distinctive brown and mottled juvenile body plumage and by the white fringes on the tips of their primaries and secondaries. Young birds that had replaced their juvenile body plumage were considered immatures if they still showed white fringing on their flight feathers. Our sample of adults likely includes some young birds that had not yet replaced all their juvenile flight feathers but that were old enough when collected to have worn away the white fringing on their flight feathers.

Growing primaries and secondaries were scored as decimal fractions of their full length, varying from 0.1 to 0.9. Missing feathers were given a score of 0.05 and new feathers replaced in the current episode of molting were scored as 1. For birds in active molt, old feathers received scores of 2, indicating that these feathers had been replaced in the previous episode of molting. We found no highly worn or faded feathers, so never assigned scores of 3, which would indicate feathers replaced two molts previously. For birds that were not growing primaries or secondaries when collected but that had two generations of feathers in their wings, each feather was scored as a 1 or 2 to indicate its replacement in the last (1) or next-to-last (2) episode of molting. These data constituted the raw data table illustrated in Rohwer [23] and are not presented in this paper.

Following the details in Rohwer [23], we added notations between each growing feather and its neighbor(s) in the raw summary to indicate the direction of feather replacement. Directionality scores of proximal, distal, or ambiguous (when adjacent growing feathers are the same length) were assigned to adjacent feather pairs in which at least one of the two feathers was growing [23]. Growing feathers that had both neighbors old were designated nodal and not given directionality scores, and growing feathers that had both neighbors new were designated terminal and not given directionality scores. In species where several adjacent feathers grow simultaneously, it may be desirable to limit directionality scoring to adjacent pairs of feathers that are both growing because these pairs will almost always be part of the same molt series. We could not do this for *H. mystacea* because the replacement of flight feathers progresses so slowly that directionality scores required comparing a growing feather with its neighbors and scoring directionality on the basis of whether the neighbor was new or old.

With this annotated molt summary table we then used various versions of the COUNTIF function in Excel to create the molt summary table. This table gives the number of growing feathers at every position, the number of nodal feathers, the number of terminal feathers, and the number of each of the three directionality scores for each adjacent pair of feathers where one or both were growing. This summary table is essential to give readers a sense of the reliability of conclusions concerning: 1) the number of molt series (defined as contiguous sets of feathers that are replaced successively and in the same direction [23]); 2) the boundaries between series; 3) the direction of replacement within series; and 4) the mode of replacement within molt series (stepwise or single wave). Where directionality predominates as proximal or distal between groups of adjacent feathers we present these values in bold type. For each directionality summary we present a sign test to assess its statistical significance. Perhaps the single most important feature of summary molt tables is the lower-most line that gives the number of growing feathers for each of the flight feathers. Confidence in interpretations of the rules of flight feather replacement depends on good samples of growing feathers at every feather position.

Finally, we followed Rohwer [23] to generate iterated summary tables that make the conclusions about the rules of feather replacement easy to see. In the iterated summary table directionality scores between adjacent feathers that are not part of the same molt series are added to the appropriate counts for nodal and terminal feathers. This creates a column of zeros between adjacent feathers that belong to different molt series. Presenting both the columns of zeros separating adjacent molt series and predominate directionality values in bold font makes the rules of flight feather replacement easier to perceive in the iterated table. The basis for inferring boundaries between molt series is presented in results.

### Measuring feather growth rate

We used the average distance between adjacent pairs of light dark growth bands in feathers to measure the daily rate of feather elongation. In most species that have been examined a single pair of growth bands represents the 24 hour foraging cycle [24]–[27]; some albatrosses are an exception because they feed mainly at dawn and dusk, and generate two short pairs of growth bands in 24 hours [28]. The assumption that each pair of growth bands represents a 24-hour cycle is justified for *H. mystacea* because they forage throughout the day.

### Source of breeding data

Breeding dates came from notations on tags for museum study skins, from egg sets in museum collections, and from collected nestlings that were still growing all their flight feathers. We did not extrapolate these dates to estimated dates of clutch initiation.

## Results

### Primary and secondary replacement rules in immatures

Table 1 summarizes the results for wing quill replacement patterns in immatures. Although we had 42 immatures that were molting primaries or secondaries, the totals for growing primaries and secondaries in Table 1 are low for many feathers. Nonetheless, the following general summary seems to apply to the first replacement of primaries and secondaries in immature *H. mystacea*.

1. Only P1 is nodal; this suggests that the primaries constitute a single molt series.
2. Without exception, the direction of replacement in the primaries is distal (*n* = 73), again suggesting that the primaries are a single replacement series (sign test *p*<0.001). Note, however, that the sample size of growing primaries is low for P8–P10, making omissive molt, wherein P8 or P9 is skipped to generate a second outer wave of primary replacement in the next episode of molt, impossible to evaluate [16],[17],[21].
3. Between secondary pairs S1/S2 and S7/S8 replacement is mostly proximal (12 cases) and seldom distal (3 cases; sign test *p* = 0.019), though sample sizes of growing feathers are poor from S3 through S8.
4. Between the secondary pairs S11/S10 and S9/S8 replacement tends to be distal, with a total of 6 cases of distal directionality and 4 cases of proximal directionality (sign test *p* = 0.374).

**Table 1:** Molt summary for primaries and secondaries in immature *Hemiprocne mystacea*.

	S11		S10		S9		S8		S7		S6		S5		S4		S3		S2		S1		P1		P2		P3		P4		P5		P6		P7		P8		P9		P10
**Raw summary data**																																									
Sum nodal	0		0		0		0		1		0		0		0		0		0		0		4		0		0		0		0		0		0		0		0		0
Sum distal		3		2		1		0		1		0		0		1		1		0		0		**5**		**10**		**7**		**9**		**15**		**13**		**9**		**4**		**1**	
Sum proximal		2		1		1		1		0		1		0		0		**4**		**6**		2		0		0		0		0		0		0		0		0		0	
Direction ambiguous		0		1		0		0		0		0		0		0		0		0		0		0		0		0		0		0		0		0		0		0	
Sum terminal	0		0		3		1		0		1		0		0		0		1		0		0		0		0		0		0		0		0		0		0		0
Number growing	1		3		4		1		1		2		0		0		1		5		2		2		6		5		2		7		8		5		4		0		1
**Iterated summary**																																									
Nodal	0		0		0		0		1		0		0		0		0		0		2		4		0		0		0		0		0		0		0		0		0
Distal replacement		3		2		1		0		1		0		0		1		1		0		**0**		**5**		**10**		**7**		**9**		**15**		**13**		**9**		**4**		**1**	
Proximal replacement		2		1		1		1		0		1		0		0		**4**		**6**		**0**		0		0		0		0		0		0		0		0		0	
Direction ambiguous		0		1		0		0		0		0		0		0		0		0		**0**		0		0		0		0		0		0		0		0		0	
Terminal	0		0		3		1		0		1		0		0		0		1		0		0		0		0		0		0		0		0		0		0		0
Number growing	1		3		4		1		1		2		0		0		1		5		2		2		6		5		2		7		8		5		4		0		1

This summary for immatures suggests that the primaries are a single molt series and are replaced distally, and that the secondaries are divided into an inner and outer molt series, with the outer replaced proximally and the inner replaced distally. The location of the break between the two secondary molt series in immatures is ambiguous because we found few specimens growing secondaries between S3 and S8 and because directionality scores were often contradictory in the innermost secondaries. Given the directionality scores, we interpret the single nodal feather at S7, which was also the only growing feather for S7, as an anomaly caused either by adventitious loss or by incorrectly assigning feather age to its neighbors, rather than as indicating the start of another molt series.

The iterated summary for the wing quills of immatures (Table 1) is little different than the raw summary table because the only break between molt series in the wing quills that we could assign is between the primaries and the outer secondaries. This requires moving the 2 directionality scores between P1/S1 to nodal for S1. These two scores were both generated by growing S1s (Table 1) and, thus indicate, not the direction of replacement within a molt series, but the fact that secondary molt in immatures initiates after the primary molt has been underway for some time. This delay in the initiation of the first molt of secondaries, relative to the first molt of the primaries, is well illustrated by seven immatures collected early in their secondary molt (growing either S1 or S2). In six of these cases the outermost growing primary was P6 (3 cases), P7 (2 cases) or P8 (1 case). In the seventh case the outermost growing primary was P3, but this bird was likely an adult that was mis-aged as an immature because it was growing two adjacent primaries simultaneously, something we occasionally saw in adults, but that occurred in no other immature.

### Primary and secondary replacement rules in adults

Table 2 summarizes wing quill replacement patterns for *H. mystacea* for the 220 adults that were molting primaries or secondaries. Recall, however, that as molt progresses to the outer-most primaries in immatures, they tend to lose the white fringing on their flight feathers; thus, some “adults” are likely to be immatures that had lost the juvenile fringing on their flight feathers. From the summary of the raw data for adults replacing primaries or secondaries (Table 2), the following patterns emerge:

1. The direction of replacement in the primaries in adults is much more distal (n = 294) than proximal (*n* = 8; sign test *p*<0.001), which is consistent with the primaries being a single molt series. The cases of proximal replacement are difficult to explain. They could represent errors in assigning feather age or they could be real if a wave of feather replacement in the inner primaries had progressed outward far enough that the growing feather in that wave was proximal to a new feather replaced in that same episode of molting by another wave of molt that was initiated in the outer primaries.
2. There are nodal feathers scattered throughout the primaries. These feathers are surrounded by feathers that we scored as old. These nodal primaries suggest that molt in adults can be interrupted for long enough periods of time that, when molt is reinitiated, the primary proximal to the point of re-initiation, which should have been replaced in the previous episode of molt, has become sufficiently worn and faded that we scored it as old.
3. Between secondary pairs S1/S2 and S7/S8 secondary replacement is much more strongly proximal (45 cases) than distal (14 cases; sign test *p*<0.001), suggesting that S1 through S7 or S8 constitutes the outer molt series in the secondaries.
4. Between the secondary pairs S11/S10 and S9/S8 secondary replacement tends to be distal, with 8 cases of distal directionality and 6 cases of proximal directionality (sign test *p* = 0.395).
5. As in the primaries, the many nodal feathers in the secondaries suggests that molt is frequently interrupted before all the secondaries are replaced in either of the two secondary molt series.

**Table 2:** Molt summary for primaries and secondaries in adult *Hemiprocne mystacea*.

	S11		S10		S9		S8		S7		S6		S5		S4		S3		S2		S1		P1		P2		P3		P4		P5		P6		P7		P8		P9		P10
Raw summary																																									
Sum nodal	0		3		1		3		0		2		2		0		2		0		1		2		0		0		0		2		6		2		4		4		9
Sum distal		1		4		3		2		1		1		3		3		3		1		1		**9**		**20**		**24**		**23**		**42**		**48**		**42**		**43**		**43**	
Sum proximal		0		2		4		3		**6**		**9**		**9**		**8**		**5**		**4**		**5**		4		3		1		0		0		0		0		0		0	
Ambiguous direction		0		0		0		1		1		0		0		0		0		0		0		0		2		0		0		0		0		0		0		1	
Sum terminal	0		0		2		2		0		3		0		1		0		2		0		2		2		0		1		0		0		1		0		2		0
Number Growing	0		4		8		9		5		10		8		7		7		5		3		9		14		14		10		15		35		22		28		27		34
Iterated summary																																									
Sum nodal	0		3		1		3		0		2		2		0		2		0		3		6		0		0		0		2		6		2		4		4		6
Sum distal		1		4		4		**0**		1		1		3		3		3		1		**0**		**9**		**20**		**24**		**23**		**42**		**48**		**42**		**43**		**43**	
Sum proximal		0		2		4		**0**		**6**		**9**		**9**		**7**		**4**		**4**		**0**		4		3		1		0		0		0		0		0		0	
Ambiguous direction		0		0		0		**0**		1		0		0		0		0		0		**0**		0		2		0		0		0		0		0		0		1	
Sum terminal	0		0		2		4.5		3.5		3		0		1		0		2		0		0		2		0		1		0		0		0		0		2		22
Number Growing	0		4		8		9		5		10		8		7		7		5		3		9		14		15		11		15		35		22		28		27		34

This summary of the raw molt scores for adults suggests that the primaries are replaced distally as a single molt series, and that the outer secondaries are a separate molt series because directionality is strongly proximal through the S6/7 feather pair. The nodal feathers scattered throughout the primaries and outer secondaries suggest interruptions of molt for substantial periods of time.

The iterated summary for adults places a break between S1 and P1. This break is supported by the switch in the direction of feather replacement between the P1/P2 and the P1/S1 feather pairs. In immatures this break was also supported by the delay in the initiation of secondary molt relative to primary molt. However, in adults the frequent interruption and reinitiation of the molt at points throughout these two series obscures any difference in the timing of initiation of feather replacement in these two series. In the iterated summary table for adults we moved the six directionality scores for the P1/S1 pair to either P1 or S1, depending on which was the growing feather. The break between the inner and outer series of secondaries probably lies between S7 and S8 because directionality is clearly distal from the pairs S1/2 through S6/7. Thus in the iterated summary we have moved the six directionality scores to terminal for either S7 or S8, depending on which was the growing feather. For the single case of ambiguous direction (S7 and S8 were both growing and the same length), we split the score as .5 to S7 and .5 to S8 (Table 2).

One aspect of primary replacement in adults might suggest that the primaries are divided into two replacement series, both of which are replaced distally. For P1–P5 the frequency of growing feathers is about half that for P6–P10, suggesting that the outer 5 primaries might be replaced more frequently than the inner 5 primaries (Table 2). Consistent with this interpretation is the fact that P6 is nodal more frequently than most other primaries (Table 2). While we think it is important to mention this as a possible alternative interpretation of the rules of primary replacement in *H. mystacea*, we also doubt that this interpretation is correct because data for immatures (Table 1) offer no suggestion that there are two series of primaries. In other species where the primaries are divided into two series, immatures initiate molt in these series independently [11],[15]. Moreover, in those species for which the ontogeny of stepwise molting is known, it develops as a single wave of feather replacement starting at P1 in immature birds, just as we see in immature *H. mystacea*, and additional waves of primary replacement develop only when the first wave of primary replacement arrests before reaching P10 [16]. We cannot explain the frequency break in the number of growing feathers that occurs in adults between P5 and P6 (Table 2), but interpreting this break as evidence that the primaries are divided into two replacement series would require that the number of molt series in the primaries changes after the first molt of primaries, which seems improbable. Erroneously aging some older immatures as adults might have contributed to this break in the frequency of feather replacement, but aging errors would be unlikely to create the sharp break in the frequency of growing primaries seen between P5 and P6 (Table 2).

### Is replacement stepwise?

Evidence for stepwise replacement of flight feathers requires showing first that the feathers being considered constitute a single molt series and, second, that in some individuals two or more waves of feather replacement progress in the same direction through the series simultaneously [16],[17]. Given that we have shown that the 10 primaries and the 7 outer secondaries each constitute single molt series, then the presence of new feathers in these series that are separated by older would be strong evidence of stepwise replacement. However, feather ages were often difficult to assign in *H. mystacea*, so we did not use this criteria. Instead, we considered only wings that had actively growing feathers, and counted the number of specimens that had two or more growing feathers that were separated by fully grown feathers, a much more conservative, but completely reliable method of assessing the frequency of stepwise flight feather replacement.

There is strong evidence of stepwise molt for adults, both in the primaries and in the outer secondaries. There were 166 adults growing primaries; 34 had two waves of primary replacement separated by fully-grown primaries, and one had three waves of primary replacement separated by fully grown primaries. Thus, 21.0% of adults that were replacing primaries had more than one wave of molt proceeding through their primaries simultaneously. There were 39 adults replacing secondaries in the outer molt series (S1–S7); five had two waves of secondary replacement separated by fully-grown secondaries. Thus stepwise replacement of the outer secondaries was seen in 12.8% of adults that were molting secondaries. If this figure for the outer secondaries is adjusted upward in proportion to the difference in the number of feathers in the outer secondary series (7) and the primaries (10), step-wise replacement would occur about 18.3% of the time, compared to 21.0% in the primaries. Although we also observed a few cases of multiple waves in the inner secondaries, we do not present similar percentages for this molt series because directional scores were so contradictory that we are unsure how S8–S11 are replaced and if they constitutes a single molt series.

### Time required to replace the primaries

Assuming no interruptions in the molt, the time required to replace all the feathers of a molt series is determined by three variables, the summed length of the feathers in the molt series, the rate at which feathers grow, and the number of feathers growing simultaneously. The average of the summed length of the 10 primaries for four adult male and four adult female *H. mystacea* was 1085.3 mm (Table 3).

**Table 3:** Primary lengths measured (in mm) for 4 adult females and 4 adult male specimens of *Hemiprocne mystacea*.

		Primary									
UWBM #	Sex	P1	P2	P3	P4	P5	P6	P7	P8	P9	P10
58752	F	55	66	78	90	107	121	140	154	162	170
63244	F	52	60	73	83	97	111	131	142	153	159
66039	F	55	64	72	88	102	118	133	153	161	163
68087	F	52	59	71	83	95	112	129	142	155	156
60252	M	55	64	76	87	100	116	134	145	153	151
63240	M	49	64	77	87	101	118	134	151	161	162
68090	M	54	68	74	85	97	109	126	135	152	151
68099	M	55	63	75	86	101	114	130	143	155	157
**Average**		53.4	63.5	74.5	86.1	100.0	114.9	132.1	145.6	156.5	158.6

The sum of the average lengths for the 10 primaries is 1085.3 mm.

We had 179 adults that were growing primaries or secondaries; 166 of these birds were growing primaries and about a third of these were also growing secondaries. We also had 13 adults that were growing secondaries but not primaries (Table 4). Because few adults grow adjacent primaries simultaneously, we suspect that most of these 13 birds were still in the process of replacing primaries, but happened to have been collected after completing the growth of one primary and before loosing the next distal primary. Of the 179 birds that were growing primaries or secondaries, only nine were growing two adjacent primaries simultaneously. Five of these nine were anomalous because the adjacent primaries either were the same length or the more distal primary was longer than the more proximal. Thus, only four out of the 179 adults growing primaries or secondaries appeared to have been growing two adjacent primaries simultaneously as a result of normal feather replacement. We included these four adults in our calculation of the time required to replace all the primaries, but excluded the five anomalous birds.

**Table 4:** Summary of primary molt data for 174 adult *Hemiprocne mystacea* that were replacing primaries or secondaries when collected (5 adults in primary molt were excluded because their molt was anomalous).

Replacing:	Number of adults
secondaries but not primaries	13
primaries with one replacement wave	128
primaries with two replacement waves	32
primaries with three replacement waves	1
Total number of growing primaries (128+64+3, by counting waves, +4 for the four adults growing two adjacent feathers in the same wave of molt)	199
Number of adults growing primaries	161
Average number of primaries growing per wing = 1.24	
Number of adults growing primaries or secondaries	174
Average number of primaries growing per wing = 1.14	

For adults that were replacing primaries, the average number of primaries growing per wing was 1.24, slightly more than 1.0 because of the 33 adults that had two or three waves of primary replacement and because of the four birds growing two adjacent primaries (Table 4). For adults that were growing primaries or secondaries, the average number of primaries growing simultaneously drops to 1.14, because of the 13 adults that were likely collected before they had lost their next primary because they were growing secondaries but not primaries (Table 4).

We measured primary growth rate as the average distance in mm between adjacent light/dark pairs of growth in 18 specimens (Table 5). For each individual we measured the distance in mm between six sequential pairs of growth bands and divided by six. The average growth rate for the primaries of these 18 birds was 2.86 mm/d.

**Table 5:** Daily growth rate of primaries and rectrices for 18 *Hemiprocne mystacea* computed as the average distance between 6 pairs of growth bands for each bird.

Specimen number	Sex	Primary growth rate(mm per day)	Rectrix growth rate(mm per day)
USNM 377660	M	2.0	2.5
USNM 405410	M	3.0	2.5
USNM 377661	M	3.5	3.0
BM 1923.9.1580	M	3.5	2.5
FMNH 153450	M	2.5	2.5
FMNH 87584	M	2.5	2.0
MCZ 167287	M	3.0	2.5
MCZ 290664	M	3.0	2.0
BM 1903.12.2.44	F	2.5	2.5
BM 1939.12.9.1681	F	3.0	2.5
MVZ 89582	F	2.5	2.5
Yale 74979	F	3.5	2.5
FMNH 153451	F	2.5	1.5
FMNH 87586	F	3.0	1.5
FMNH 87583	F	2.5	2.0
FMNH 302623	F	3.0	2.5
FMNH 302625	F	3.5	2.0
MCZ 167286	F	2.5	1.5

Assuming no interruptions of the primary molt, the number of days that an average adult *H. mystacea* would take to replace all of its primaries is given by
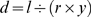
where *d* is days, *l* is the summed length of the primaries, *r* is primary growth rate in mm/d, and *y* is the average number of primaries growing simultaneously. When only adults that were growing primaries are used to estimate *y*, the primary molt is estimated to require 306 days. If the 13 adults that were molting secondaries but not primaries are included in the estimate of *y*, the estimated duration of the primary molt increases to 333 days. The latter figure is likely closer to reality because it corrects for the probable pause between completing the growth of one primary and loosing the next (see above). Because many more birds were molting primaries than secondaries (Table 1), our estimate of *y* may still be somewhat high, suggesting that 333 days may still underestimate the average time adults spend replacing all their primaries in an uninterrupted molt.

### Replacement of the rectrices

We examined only six juvenile specimens of *H. mystacea* that were molting rectrices. Three of these immatures were initiating rectrix molt, and all were replacing R5, the outermost rectrix. For two of these specimens the outermost growing primary was 6 or 8; for the third no primary was growing but P1–6 were new and P7–10 were old. Apparently, rectrix replacement starts late enough in the first molt of primaries that the white fringing on the flight feathers has been lost in most birds before rectrix molt is initiated. For this reason, our sample of adults molting rectrices probably includes some immatures.

Table 6 summarizes rectrix replacement for the 58 presumed adults that were growing rectrices. From the raw data summary the following patterns emerge:

1. R5 is frequently nodal and the direction of replacement between R4 and R5 is often contradictory (11 distal, 7 proximal; sign test *p* = 0.24). Together these data suggest that R5 constitutes its own molt series.
2. The direction of replacement from R4 to R1 is much more strongly proximal (*n* = 34) than distal (*n* = 9; sign test *p*<0.001), suggesting that R4 through R1 are a single molt series.
3. Finally, most of the rectrices received at least occasional scores as nodal or terminal, suggesting that the rectrix molt may be interrupted, as we also found for the primaries and secondaries.

**Table 6:** Molt summary for the rectrices in adult *Hemiprocne mystacea*.

	R1		R2		R3		R4		R5
**Raw summary data**									
Sum N	2		0		0		3		13
Sum distal		1		4		4		11	
Sum proximal		**13**		**11**		**10**		7	
Ambiguous		0		0		1		0	
Sum T	0		1		0		0		0
Number Growing	11		8		11		11		23
**Iterated summary**									
Sum N	2		0		0		9		13
Sum distal		1		4		4		**0**	
Sum proximal		**13**		**11**		**10**		**0**	
Ambiguous		0		0		1		**0**	
Sum T	0		1		0		2		10
Number Growing	11		8		11		11		23

Generating the iterated summary (Table 6) that reflects two molt series in the rectrices, requires moving all the directionality scores between R4/5 to either nodal or terminal for R4 or R5. To do this we used the following rules: 1) If R5 was growing and R4 was old, then the implied proximal replacement was changed to nodal for R5. 2) If R5 was growing and R4 was new, then the implied distal replacement was changed to terminal for R5. 3) If R4 was growing and R5 was new, then the implied proximal replacement was changed to nodal for R4 if R3 was old. 4) If R4 was growing and R5 was new, then the implied distal replacement was changed to terminal for R4 if R3 was new. This last case may be a consequence of error in scoring the age of R3 because R4 seems to be nodal for the inner series of rectrices, so R3 should always be older than R4.

With these changes the iterated summary for replacement in the rectrices looks reasonable (Table 6). R5 scores as nodal about as frequently as it scores as terminal, reflecting its status as a separate series, as was suggested by the frequent contradictions in the direction of replacement for R4/5 in the raw summary table. R4 becomes frequently nodal, reflecting the fact that it should be the first rectrix of the inner molt series to be replaced when molt in this series is initiated for the first time in immatures or molt is reinitiated after a complete replacement of the rectrices in adults.

In most specimens just a single rectrix was growing per side, but in three of the 23 specimens with R5 growing an inner rectrix was also growing; thus, the two rectrix molt series are sometimes activated simultaneously. We also looked for evidence of multiple waves of rectrix replacement in the inner rectrices. For three birds at least two feathers in this series were either missing or growing simultaneously, but all were abnormal. In all three of these cases one or two feathers were missing that were likely lost when the bird was collected or prepared. In another, which was also one of the three cases just mentioned, two adjacent rectrices were nearly fully grown, suggesting that they may have been lost at the same time accidentally, before the specimen was collected. Thus there is no credible evidence of stepwise replacement for R1–4.

### Schedules of molt and breeding

**Figure 1:**
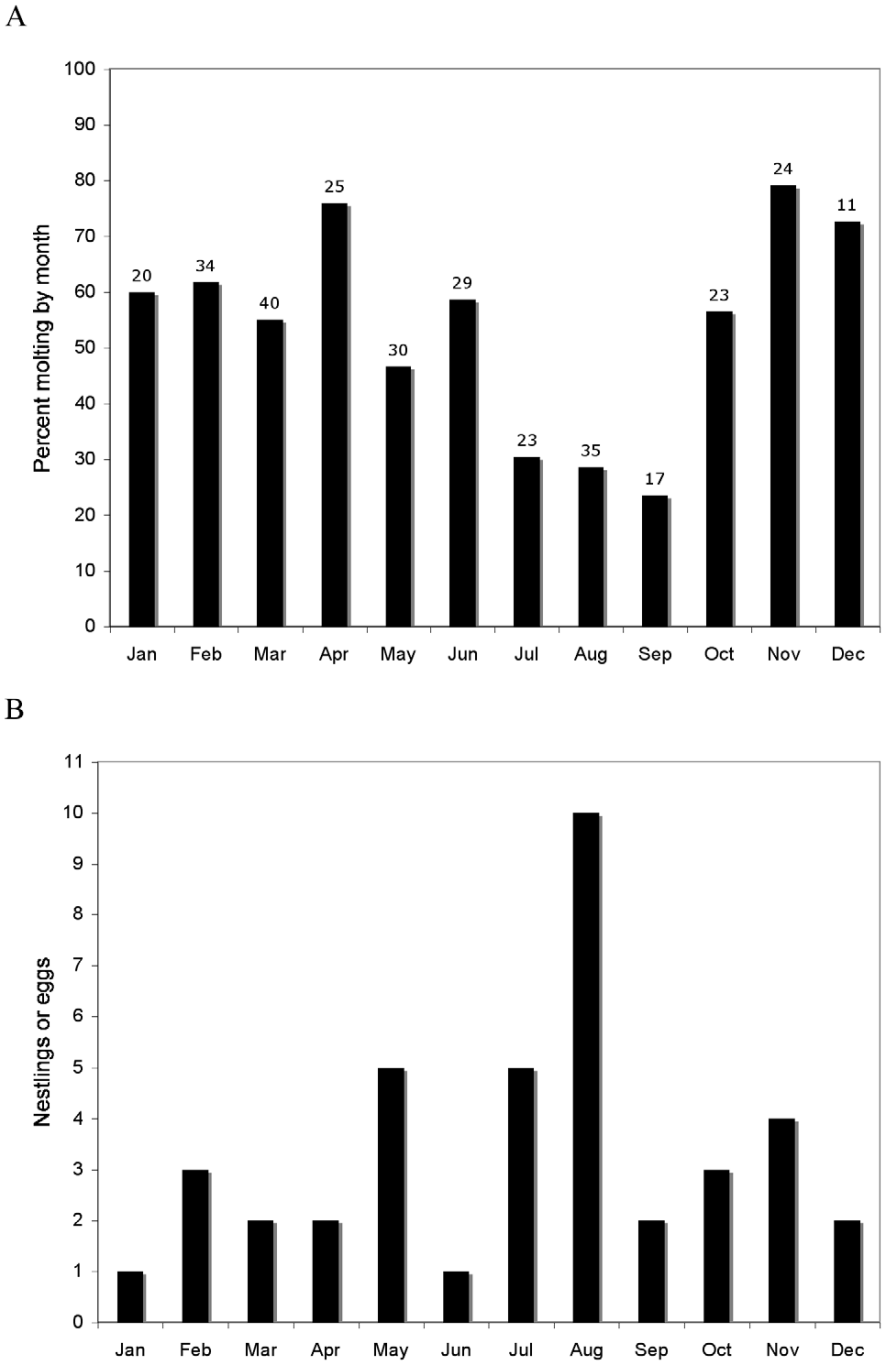
Overlap of molt and breeding in *Hemiprocne mystacea* at the population level. A is the monthly percentage of birds molting primaries (numbers over bars are sample sizes). B is the number of active nests per month for the 40 breeding records we could find.

Figure 1 summarizes the seasonal distribution of birds molting primaries and of birds involved in parental care. At the population level molt and breeding occur in every month of the year. Because the molt of primaries requires more than 300 days there is little doubt that individual birds often replace flight feathers while they are caring for their single egg or small young, and we have four cases of adults shot from nests that were molting primaries (USNM 572397; UWBM 67987, 68099 and 68107). The fraction of adults that are replacing primaries is lowest in the months of July, August and September, and the number of the breeding records we have is correspondingly highest in two of these three months, suggesting that individuals may interrupt primary molt when the demands of parental care are highest (Fig. 1). This inverse relationship between the fraction of adults replacing primaries and nest counts is just significant (Mann-Whitney test; U = 2; N_1_ = 3, N_2_ = 9; *p* = 0.05), but the groups were suggested by the data, so the significance value cannot be trusted.

While data from specimens examined at just a single point in time cannot directly demonstrate the interruption of primary molts, we can infer arrests in primary replacement from the ontogenetic mechanism for developing multiple waves of feather replacement. In all species for which the ontogeny of step-wise molting been studied, it develops as a consequence of molt being arrested before a wave of feather replacement reaches the terminal feather in its molt series [16]. Then, in the next episode of molting, feather replacement starts again both where it was arrested in the preceding molt and at the first feather in the series. Assuming the same ontogeny in *H. mystacea*, we can infer that the lower frequency of molting birds in July, August and September reflects arrests in primary replacement.

## Discussion

There are two important messages in this paper. The first is that the biology of flight feather replacement in *H. mystacea* has many interesting life history implications. The second is that the tables we use to summarize molt data from hundreds of specimens should succinctly and clearly allow others to assess both the validity of our interpretations and whether those interpretations are based on adequate data. Hopefully, the iterated versions of these tables will make our interpretations of the patterns of flight feather replacement more easily accessible. These tables should speak for themselves, so we offer no further discussion of them.

Complex primary replacement has been reported for more than 200 species of birds that weigh less than 100g, but almost all of these are small parrots, falcons, and kingfishers in which the primaries are divided into two or more molt series [10]. To our knowledge, *H. mystacea* is the first small species studied well enough to be certain that the primaries constitute a single molt series. Although *Apus apus* sometimes replaces P10 while inner primaries are growing [29], these data for *H. mystacea* appear to be the first clear demonstration of stepwise primary replacement occurring throughout the wing in a small bird. We also show that the secondaries are divided into at least two molt series and that replacement in the outer secondary series (S1–7) can also be stepwise. As documented for Western Kingbirds, *Tyrannus verticalis* [23] and Rough-winged Swallows, *Stelgidopterix ruficollis* [30], *H. mystacea* also replaces its rectrices in two molt series, with the outermost rectrix constituting a unique series, even though the outermost rectrix is R6 in the former two species and R5 in *H. mystacea*. Unlike these (and other) passerines, however, *H. mystacea* replaces its inner rectrices proximally, rather than distally.

The primaries of *H. mystacea* are estimated to grow 2.86 mm/d based on measures of growth bands, and this rate falls close to the expected value for a 60g bird [10]. The computed time required to replace all the primaries without interruption likely exceeds 333 days, which may be the slowest primary replacements described for any bird relative to its body size. For adult *H. mystacea* that were replacing wing quills, the mean number of primaries growing per wing was only 1.14 feathers. Non-aerial foragers of similar size often grow 3–4 primaries simultaneously [9]. Growing 1.14 primaries per wing is a very low number, and especially low for a species with stepwise primary replacement, because stepwise primary replacement is thought to increase the rate of primary replacement while minimizing the aerodynamic costs of missing feathers [31],[32].

Primary replacement in *H. mystacea* is probably exceedingly slow for two reasons. First, aerial foragers replace their primaries very slowly relative to other birds of similar size, as shown by data on the duration of the primary molt in two species of swallows [29],[30] and by these data for *H. mystacea*. Second, species that overlap molt and breeding typically have less than two primaries growing per wing, which greatly prolongs primary replacement [9]. The data we could assemble on molt breeding overlap in *H. mystacea* clearly show that molt and breeding overlap extensively at the population level, and four adults shot at nests were molting primaries when collected. However, documenting that interruptions of molt are associated with high demands of parental care will require following marked individuals. Interruptions in the replacement of primaries and outer secondaries likely provide the mechanism for generating multiple waves of feather replacement that are sometimes observed in these two molt series [16],[17].

Stepwise molting has the disadvantage of replacing the inner primaries, which wear the least, more frequently than outer primaries, which wear the most [16],[17]. In a few well-studied species with stepwise primary replacement, some immatures skip an outer primary (referred to as omissive molts [21]), apparently to set up a point of molt initiation in the outermost primaries for the following molt. Omissive molts help overcome the failure of stepwise molts to replace the outer juvenile primaries as quickly as they wear out [16], [17]. We found no evidence of omissive molt of the juvenile primaries in *H. mystacea*, but we examined so few juvenile *H. mystacea* replacing outer primaries that we cannot safely infer its absence.

Stepwise replacement of the primaries has been reported in a great diversity of avian lineages, mostly involving large species [18],[33]. It has likely evolved many times as a mechanism for generating multiple waves of replacement that can rapidly replace feathers when the time available for molting is unpredictable due to breeding failures or variable food supplies [17],[19]. The transition from replacing the primaries in a single-wave (which predominates in small birds [10]) to stepwise replacement, requires only that, following arrests in molt, primary replacement reinitiates both wherever it was arrested and at P1. This seems like a simple evolutionary transition compared to a transition to two independently activated molt series in the primaries.

Primary replacement in *H. mystacea* is so exceedingly slow that arguing that stepwise molting evolved to facilitate rapid primary replacement is implausible. Instead, it may be a consequence of the ontogeny of stepwise flight feather replacement. In 13 species for which data on the mode of development of stepwise molting has been summarized, immatures started their first primary replacement at P1 and did not develop a second wave of primary replacement until the first episode of molt was interrupted [16]. Then, when molt reinitiated, these species picked up primary replacement with the next feather to be replaced in the first wave of molt and started again at P1. If this is how multiple waves of feather replacement develop in *H. mystacea*, stepwise replacement of the primaries and secondaries may be a consequence of interruptions of flight feather replacement caused by the demands of parental care or by poor environmental conditions for molting, rather than an adaptation to reduce the time in molt. When stepwise molting evolves to reduce the time in molt, we expect to see multiple primaries at several spaced-out loci growing simultaneously [31]. But in *H. mystacea*, the number of primaries growing simultaneously is lower than has been recorded for any other bird, including another aerial forager with simple descendent replacement of the primaries [30]. The very slow progression of flight feather molt in *H. mystacea* suggests that the occasional stepwise replacement found in its the primaries and outer secondaries could be an accident of history and ontogeny in this remarkable bird.

## References

[1] Lindstrom A, Visser GH, Daan S (1993). The energetic cost of feather synthesis is proportional to basal metabolic rate.. Physiol Zool.

[2] Murphy ME, Taruscio TG (1995). Sparrows increase their rates of tissue and whole-body protein-synthesis during the annual molt.. Comp Biochem Physiol A.

[3] Tucker VA (1991). The effect of molting on the gliding performance of a Harris Hawk (*Parabuteo unicinctus*).. Auk.

[4] Chai P (1997). Hummingbird hovering energetics during moult of primary flight feathers.. J Exp Biol.

[5] Lind J (2001). Escape flight in moulting tree sparrows (*Passer montanus*).. Funct Ecol.

[6] Slagsvold T, Dale S (1996). Disappearance of female Pied Flycatchers in relation to breeding stage and experimentally induced molt.. Ecology.

[7] Swaddle JP, Witter MS, Cuthill IC, Budden A, McCowen P (1996). Plumage condition affects flight performance in common starlings: Implications for developmental homeostasis, abrasion and moult.. J Avian Biol.

[8] Jenni L, Winkler R (1994). Moult and Ageing of European Passerines.

[9] Rohwer VG, Rohwer S, Ortiz-Ramirez MF (2009). Molt biology of resident and migrant birds of the monsoon region of west Mexico.. Ornithol Neotrop.

[10] Rohwer S, Ricklefs RE, Rohwer VG, Copple MM (2009). Allometry of the duration of flight feather molt in birds.. PLoS Biol.

[11] Langston NE, Rohwer S (1995). Unusual patterns of incomplete primary molt in Laysan and Black-footed Albatrosses.. Condor.

[12] Edwards AE, Rohwer S (2005). Large-scale patterns of molt activation in the flight feathers of two albatross species.. Condor.

[13] Forshaw JM, Cooper WT (1977). Parrots of the world.

[14] Miller AH (1941). The significance of molt centers among the secondary remiges in the Falconiformes.. Condor.

[15] Pyle P (2005). Remigial molt patterns in North American Falconiformes as related to age, sex, breeding status, and life-history strategies.. Condor.

[16] Rohwer S (1999). Time constraints and moult-breeding tradeoffs in large birds.. Proc Int Ornithol Congr.

[17] Shugart GW, Rohwer S (1996). Serial descendant primary molt or Staffelmauser in Black-crowned Night-herons.. Condor.

[18] Stresemann E, Stresemann V (1966). Die Mauser der Vögel.. J Orn.

[19] Filardi CE, Rohwer S (2001). Life history implications of complete and incomplete primary molts in Pelagic Cormorants.. Condor.

[20] Potts GR (1971). Moult in the shag *Phalacrocorax aristotelis* and the ontogeny of the “staffelmauser”.. Ibis.

[21] Rasmussen PC (1988). Stepwise molt of remiges in Blue-eyed and King Shags.. Condor.

[22] Edelstam C (1984). Patterns of moult in large birds of prey.. Ann Zool Fenn.

[23] Rohwer S (2008). A primer on summarizing molt data for flight feathers.. Condor.

[24] Murphy ME, King JR (1991). Ptilochronology: A critical evaluation of assumptions and utility.. Auk.

[25] Wood HB (1950). Growth bars in feathers.. Auk.

[26] Brodin A (1993). Radio-ptilochronology – tracing radioactively labelled food in feathers.. Ornis Scand.

[27] Michner H, Mivhner JR (1938). Bars in flight feathers.. Condor.

[28] Langston NE, Rohwer S (1996). Molt-breeding tradeoffs in albatrosses: Life history implications for big birds.. Oikos.

[29] Ginn HB, Melville DS (1983). Moult in Birds.

[30] Yuri T, Rohwer S (1997). Molt and migration in the Northern Rough-winged Swallow.. Auk.

[31] Ashmole N (1968). Breeding and molt of the white tern (*Gygis alba*) on Christmas Island, Pacific Ocean.. Condor.

[32] Stresemann V, Stresemann E (1960). Die Handschwingenmauser der Tagraubvögel.. J Orn.

[33] Bridge ES (2007). Influence of morphology and behavior on wing-molt strategies in seabirds.. Mar Ornithol.

